# Transcriptomic Study for Identification of Major Nitrogen Stress Responsive Genes in Australian Bread Wheat Cultivars

**DOI:** 10.3389/fgene.2020.583785

**Published:** 2020-09-30

**Authors:** Nigarin Sultana, Shahidul Islam, Angela Juhasz, Rongchang Yang, Maoyun She, Zaid Alhabbar, Jingjuan Zhang, Wujun Ma

**Affiliations:** ^1^State Agriculture Biotechnology Centre, College of Science, Health, Engineering and Education, Murdoch University, Perth, WA, Australia; ^2^School of Science, Edith Cowan University, Joondalup, WA, Australia

**Keywords:** transcriptomics, nitrogen use efficiency, Australian wheat cultivars, nitrogen stress, RNA-seq

## Abstract

High nitrogen use efficiency (NUE) in bread wheat is pivotal to sustain high productivity. Knowledge about the physiological and transcriptomic changes that regulate NUE, in particular how plants cope with nitrogen (N) stress during flowering and the grain filling period, is crucial in achieving high NUE. Nitrogen response is differentially manifested in different tissues and shows significant genetic variability. A comparative transcriptome study was carried out using RNA-seq analysis to investigate the effect of nitrogen levels on gene expression at 0 days post anthesis (0 DPA) and 10 DPA in second leaf and grain tissues of three Australian wheat (*Triticum aestivum*) varieties that were known to have varying NUEs. A total of 12,344 differentially expressed genes (DEGs) were identified under nitrogen stress where down-regulated DEGs were predominantly associated with carbohydrate metabolic process, photosynthesis, light-harvesting, and defense response, whereas the up-regulated DEGs were associated with nucleotide metabolism, proteolysis, and transmembrane transport under nitrogen stress. Protein–protein interaction and Kyoto Encyclopedia of Genes and Genomes (KEGG) pathways analysis further revealed that highly interacted down-regulated DEGs were involved in light-harvesting and photosynthesis, and up-regulated DEGs were mostly involved in steroid biosynthesis under N stress. The common down-regulated genes across the cultivars included photosystem II 10 kDa polypeptide family proteins, plant protein 1589 of uncharacterized protein function, etc., whereas common up-regulated genes included glutamate carboxypeptidase 2, placenta-specific8 (PLAC8) family protein, and a sulfate transporter. On the other hand, high NUE cultivar Mace responded to nitrogen stress by down-regulation of a stress-related gene annotated as beta-1,3-endoglucanase and pathogenesis-related protein (PR-4, PR-1) and up-regulation of MYB/SANT domain-containing RADIALIS (RAD)-like transcription factors. The medium NUE cultivar Spitfire and low NUE cultivar Volcani demonstrated strong down-regulation of Photosystem II 10 kDa polypeptide family protein and predominant up-regulation of 11S globulin seed storage protein 2 and protein transport protein Sec61 subunit gamma. In grain tissue, most of the DEGs were related to nitrogen metabolism and proteolysis. The DEGs with high abundance in high NUE cultivar can be good candidates to develop nitrogen stress-tolerant variety with improved NUE.

## Introduction

Over the past several decades, application of nitrogen fertilizer has been a practiced way to gain optimal crop yield. N fertilizer usage is predicted to reach 105 Tg N by 2030 and 135 Tg N by 2050 (Good et al., 2004). However, overuse of fertilizers can cause significant environmental issues such as erosion, soil quality depletion, and contamination of water supplies at local, regional, and global scales (Ahmad et al., 2008; Guo et al., 2010). Thus, it is important to develop new varieties with high nitrogen use efficiency (NUE). A better understanding of gene expression and regulation under nitrogen stressed conditions will help achieve this goal. Response to nitrogen scarcity in plants is controlled by changes in gene expression involved in different molecular mechanisms that are mainly related to plant developmental processes and yield (Zhang et al., 2006; Kant et al., 2011).

In particular, wheat grain production largely depends on the provision of N fertilizer and cultivars with high N uptake and utilization efficiency (Nyikako et al., 2014; Garnett et al., 2015; Cormier et al., 2016; Hitz et al., 2017). The biological pathways related to NUE are known to be strongly influenced by genetic variation as well as environmental factors such as N availability (Moll et al., 1982; Xu et al., 2012). Studies showed that N limitation can negatively affect wheat growth, morphology, and agronomic traits (Chandna and Ahmad, 2015; Curci et al., 2017; Wen et al., 2018; Wang J. et al., 2019).

Identifying key genes to improve stress tolerance in low N conditions is a feasible way to raise NUE. It is important to select cultivars that have contrasting NUEs for a comparative understanding of gene expression and regulation in response to N stressed conditions (Hirel et al., 2007; Kant et al., 2010). There are a number of approaches that have been undertaken by researchers to unravel how plants adapt to stressed conditions (Shrawat et al., 2008). In recent years, next-generation sequencing techniques have provided opportunities to study the gene expression and their regulations at the transcriptome level, and they have significantly enhanced the success rate of gene discovery (Diao et al., 2019). A number of studies also reported on transcriptome profiling by using Illumina’s RNA-sequencing (Dai et al., 2015). Most of the studies demonstrated how a single genotype performed using contrasting environmental and growth conditions. In Arabidopsis, N response-related genes were identified using microarray analysis of gene expression changes in response to short-term and long-term treatments for nitrate with different concentrations (Wang et al., 2001; Price et al., 2004). Likewise, transcriptome study on different tissues with short-term N stress in rice also revealed a significant number of N responsive genes (Lian et al., 2006). Transcriptome study on long-term N stress was also reported in rice (Ym et al., 2009). However, a comprehensive transcriptome investigation by combining contrasting tissue, developmental stage, genotype, and N treatment is still lacking.

Nitrogen stress has a significant impact on the overall plant physiological process (Zhao et al., 2005) related to plant height, dry matter, grain yield (GY), and grain protein content (GPC) (Baligar et al., 2007; Wang W. et al., 2003). Nitrogen strongly influences photosynthesis through a large deposition of leaf N to ribulose bisphosphate carboxylase/oxygenase (Rubisco) and its involvement in stomatal opening (Evans, 1989). Approximately 75% of leaf N is allocated to chloroplasts, with about 27% of this utilized in Rubisco to ensure high photosynthetic activity (Evans, 1989; Makino, 2003). Nitrogen also influences photosynthesis via its impact on CO_2_ assimilation and sugar partitioning (Drew et al., 1989; Foyer et al., 2011; Ishikawa-Sakurai et al., 2014). The decreased photosynthesis ultimately resulted in decreased biomass production and yield (Poorter and Evans, 1998; Long et al., 2006; Jin et al., 2015).

The regulation of plant photosynthetic activity is reported to be associated with brassinosteroids (BRs), a class of steroid hormones (Sakamoto et al., 2006; Komatsu et al., 2010). BRs are known to regulate stress responses and play important roles in regulating plant growth and development (Wang et al., 2012; Zhao and Li, 2012; Hayes, 2019). Several studies in Arabidopsis and rice showed involvement of BRs in controlling flowering, leaf senescence, chloroplast development, plant height, tiller numbers, and biomass, which are important agronomic traits affecting GY (Chono et al., 2003; Mussig et al., 2003; Sakamoto et al., 2006; Wu et al., 2008; Jeong et al., 2010). In wheat, BRs were also reported to be involved in promoting root growth and water stress tolerance (Hayes, 2019; Hou et al., 2019). However, the correlation of N stress on steroid biosynthesis has not been well studied. Thus, response to N stress is a rather complex process, and a better understanding of genes involved in different pathways is needed to develop stress-tolerant wheat varieties.

This study investigated three Australian bread wheat varieties, Mace, Spitfire, and Volcani, which are known to have high, medium, and low NUEs, respectively (Alhabbar et al., 2018b). Since gene expression in plants is controlled in a temporal and tissue-specific manner (Koltunow et al., 1990; Maizel and Weigel, 2004) and the N demand is subject to plant developmental stages, the current study used different tissues at different growth stages to unravel the broad picture of transcriptome profile with the objectives of identifying novel genes that are differentially expressed under long-term N stress compared to high N treatment, and by characterizing the underlying physiological and molecular mechanisms of tolerance to N stress.

## Materials and Methods

### Plant Material, Growth Conditions, and Sample Collection

Three Australian wheat cultivars, Mace, Spitfire, and Volcani, were used in this study. Plants were grown in a glasshouse with a complete randomized block design (RCBD) including three replicates and using pots (190 mm height × 200 mm top diameter × 180 mm bottom diameter) without holes to avoid leaching. Plants were grown under controlled temperature and sunlight conditions [20/11°C (day/night)] for an 8 h light and 16 h dark photoperiod. The pots were watered manually based on soil water capacity. All plants were supplied with a basal nitrogen dose of 25 kg N ha^–1^ after 1 week of sowing. Nitrogen-free Hoagland solution^1^ was applied to all plants once every 2 weeks to meet the nutrient demand of plants except N. Two N rates—low (LN)/0 kg N ha^–1^ and high (HN)/100 kg N ha^–1^—were applied at mid-tillering (Zadoks scale 22–25) and booting (Z43–Z45) stages for plants considered as low and high N treated, respectively. The timing for N applications was adjusted according to Zadoks (Z) decimal growth stage for wheat. Flexi-N (containing 50% urea, 25% nitrate, and 25% ammonium) was used as a source of N because of its high N content (42.2% N). Flexi-N was used since it contains nitrate that is directly available to plants while the urea and the ammonium become available more slowly, enabling a controlled release of N over an extended period (CSBP, 2012). Times for N application, recording of flowering time, measurement of chlorophyll content, and tissue collection were adjusted according to each cultivar’s growth stage. For RNA extraction, the whole flag and second leaf samples were collected at the start of the flowering [0 days post anthesis (DPA)], 10, 20, and 30 DPA, while the developing grains were collected at 10, 20, and 30 DPA from the middle section of the main head, then snap-frozen in liquid nitrogen, and then stored at −80°C for later RNA extraction. Anthesis dates were estimated by the appearance of anthers on approximately 50% of all heads. Plant height was measured from soil surface to the top of the plant, and peduncle length was measured from the peduncle bottom to the joint with the stem. Chlorophyll content was measured using a handheld chlorophyll meter (IC-CCM-200—Chlorophyll Concentration Meter CCM-200 plus). One value per plant was taken from the flag leaf and second leaf on the main stem at four different growth stages: flowering (0 DPA), 10, 20, and 30 DPA. Each value was the average of three measurements recorded from the middle of the leaves. The main stem of each plant was individually labeled to ensure the same leaves were always measured. All plants in a pot (main stem plus tillers) were hand-harvested to measure yield components and the head number per plant counted. The heads were cut off and the seed number per head was counted. Grain samples were oven-dried in a forced-air circulating dryer at 60°C for 72 h. GPC was measured by near-infrared reflectance (NIR) spectroscopy using a FOSS NIR Systems model 5000 spinning cup. NIR data collection used DPIRD wheat calibrations developed over many years with the WinISI software (FOSS NIR Systems Inc., Laurel, MD, United States).

### RNA Isolation, Library Construction, and Sequencing

Leaf and grain samples from three biological replicates were ground in liquid nitrogen, and the total RNA was extracted using a pre-chilled Trizol reagent (Invitrogen, Carlsbad, CA) following the manufacturer’s directions, with some modifications. Proteins were removed with a protein extraction buffer (1 M Tris–HCl, 5 M NaCl, 10% SDS, 0.125 M EDTA, and 1 M DTT). After the protein removal, the acid phenol/chloroform/isopropanol (49:49:2), Trizol, and chloroform were added sequentially for the extraction of total RNA. Isopropanol was used for the precipitation of total RNA, which was subsequently treated with the Qiagen DNase kit to remove potential genomic DNA contamination. Concentration and purity were checked by Nanodrop, with 260/280 absorbance ratios of approximately 2.0, and the degradation and potential contamination was tested by agarose gel electrophoresis. RNA integrity was confirmed with an Agilent 2100 Bioanalyzer (Agilent Technologies, Palo Alto, CA). The mRNA was enriched using oligo (dT) beads and then fragmented randomly in a fragmentation buffer, followed by cDNA synthesis using random hexamers and reverse transcriptase. After first-strand synthesis, a custom second-strand synthesis buffer (Illumina) was added together with dNTPs, RNase H, and *Escherichia coli* polymerase I to generate the second strand by nick-translation. The final cDNA library was ready after a round of purification, terminal repair, A-tailing, ligation of sequencing adapters, size selection, and PCR enrichment. Library concentration was first estimated using a Qubit 2.0 fluorometer (Life Technologies) and then diluted to 1 ng μl^–1^ before checking the insert size on an Agilent 2100 Bioanalyzer. The concentration was then quantified at greater accuracy by quantitative PCR (Q-PCR) (library activity >2 nM). Each library with an individual barcode was sequenced by Illumina HiSeqTM PE125/PE150 (Illumina Inc., United States).

### Transcriptome Analysis

A total of 90 different samples, including 30 each from three cultivars, Mace, Spitfire, and Volcani, were used for RNA-seq analysis. The samples were subjected to low and high nitrogen treatments to study a broad range of cell responses under nitrogen stress. For both treatment conditions, the replicates showed a high correlation coefficient (*r* > 0.8) between samples. For the RNA-seq downstream analysis, three samples (VAScLNR3, VEScHNR1, and SEScLNR2) were excluded due to sample quality. A total of 2070.85 million raw reads were filtered. A total of 1963.99 million clean reads were aligned against IWGSC RefSeq v1.0 gene models that produce 1750.09 million total mapped reads (TMRs), of which 128.89 million were mapped to multiple sites (MMR) and 1621.21 million were uniquely mapped. Among the TMRs, 810.66 million were mapped with a positive strand and 810.55 million were mapped with a negative strand (Supplementary Table 1). The average leaf Q20, Q30, and GC (Base G + Base C) contents were 96.93, 92.31, and 57.21%, respectively. Similarly, the average grain Q20, Q30, and GC (Base G + Base C) contents were 96.78, 92.16, and 57.79%, respectively. For both tissues, 95% of the total reads were filtered as cleaned reads, which confirms the fine quality of the sequencing results. Approximately, an average of 89% of clean reads were mapped for N-treated leaf samples, whereas 86% were mapped for grain tissue (Supplementary Table 2). For each sample, the percent of reads mapped to exon regions was above 90%, intron reads less than 5%, and intergenic reads less than 3%. The distribution of mapped reads of each sample in chromosome 3B was the highest, while the lowest reads were mapped in chromosome 6A. The gene expression level was measured by calculating the reads mapped to exons. Read count was proportional to the actual expression level as well as to the gene length and the sequencing depth. In order to make comparable gene expression levels estimated from different genes and experiments, fragment per kilobase of transcript per million mapped reads (FPKM) was used for normalization. Considering the influence of various gene lengths and sequencing intensity, FPKM is commonly used to make comparison of gene expression levels among different samples.

### Analysis of Differentially Expressed Genes

For the FPKM, a value of 1.0 was set as the threshold for determining whether a gene was expressed or not. HiSeq v0.6.1 (a Python package for high-throughput sequencing data analysis) was used to analyze gene expression levels in this experiment using the union mode. The correlation between samples was justified by the square of the Pearson correlation coefficient. The DESeq (version 1.10.1, R Bioconductor package) was used to conduct the differential expression analysis. The normalized data were fitted to a negative binomial generalized linear model. The threshold of the *p*-value after normalization (padj, q-value) was set as ≤0.05 for filtering accurate differentially expressed genes (DEGs). The clustering of DEGs was analyzed based on the FPKM value with the use of ggplot2 (version 2.1.0) and pheatmap (version 1.0.8) (Anders and Huber, 2010, 2012; Robinson et al., 2010; Trapnell et al., 2012). The DEGs were identified using the functional annotations of the IWGSC RefSeq v1.0 gene annotation.

### Gene Ontology and Kyoto Encyclopedia of Genes and Genomes Pathway Enrichment Analysis of DEGs

Gene ontology (GO) analysis was performed using ShinyGO v0.61^2^. GO with a false discovery rate (FDR) corrected at *p* ≤ 0.05 was regarded as significant enrichment (Young et al., 2010). KOBAS (version 2.0^3^, a web server for annotation and identification of enriched pathways and diseases, was applied for Kyoto Encyclopedia of Genes and Genomes (KEGG^4^) pathway enrichment analysis. Pathways with an FDR corrected at *p* ≤ 0.05 were considered as significant enrichment (Mao et al., 2005; Kanehisa et al., 2007).

### Protein–Protein Interaction Analysis

To predict the interaction of DEGs at the protein level under N stress and further confirmation of association of DEGs with biological pathways at the protein level, deduced amino acid sequences of DEGs were used to make a protein–protein network using the STRING (version 11.0) tool^5^, a database for known and predicted protein interactions and functional associations predicted in common pathways. Due to the lack of detailed annotation of the wheat protein data available in STRING, we used two well-annotated species, rice, and Arabidopsis, as the reference to get protein–protein interaction information of the homologous wheat proteins. The global network graph of these interactions was constructed using the experimentally evident interacted proteins, and disconnected nodes (proteins) were removed to show the advanced view of highly connected proteins. MCL clustering using the inflation parameter 1.70 was used to show the association of clusters in KEGG pathways.

### Hierarchical Cluster Analysis

Hierarchical cluster analysis was performed using the Morpheus package^6^ Complete linkage analysis was performed using the Spearman rank correlation values.

### Statistical Analysis

All data generated from the glasshouse experiments were analyzed by SPSS (version 24). Univariate analysis of variance (UNIANOVA) was used to determine the significance of different factors on each agronomic trait and protein parameter. The significant statistical difference was judged at *p* ≤ 0.05.

## Results

### Agronomic Performance of Wheat Cultivars Under Low and High Nitrogen Conditions

**FIGURE 1:**
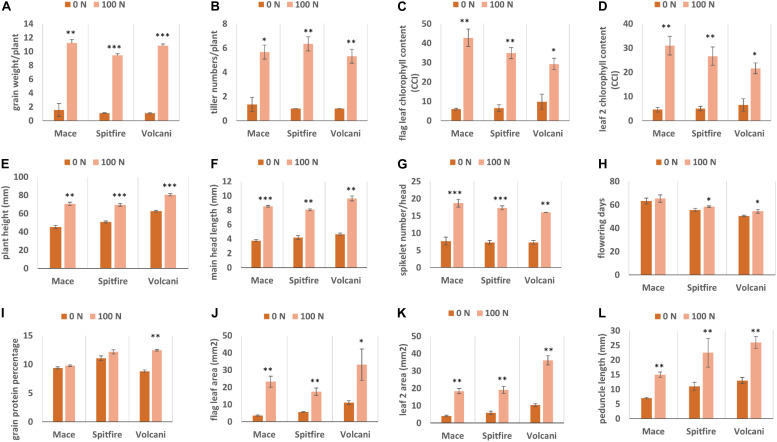
Growth and agronomic traits of *Triticum aestivum* cultivars (Mace, Spitfire and Volcani) under low (0N) and high (100 N) treatments. **(A)** grain weight/plant **(B)** tiller numbers/plant **(C)** flag leaf chlorophyll content **(D)** leaf 2 chlorophyll content **(E)** plant height **(F)** main head length **(G)** spikelet number/head **(H)** flowering days **(I)** grain protein percentage **(J)** flag leaf area **(K)** leaf 2 area **(L)** peduncle length. The values are presented as means ± standard deviation (SD) of three independent biological repeats. Error bars were calculated from three biological replicates and one-way ANOVA was used to test for significance of nitrogen treatment effects on different parameters at *P*≤0.05 level. ^∗^, ^∗∗^, ^∗∗∗^ Significant at the 0.05, 0.01 and 0.001 probability level, respectively.

Under N stress (0 Kg N/ha), most of the agronomic traits were affected negatively in all three cultivars. In general, tiller number, GY, and chlorophyll content were mostly affected by N stress, whereas flowering days and GPC were less affected. A strong variation in grain weight per plant has been observed, which is considered as a yield component for small-scale glasshouse experiments. Grain weight per plant was dropped by 78% in Mace, 81% in Spitfire, and 80% in Volcani (Figure 1A) due to N stress. Similarly, under N stress, the tiller number (Figure 1B) was decreased by 72.4, 84.2, and 81.2%, and the chlorophyll content of both flag leaf and leaf 2 (Figures 1C,D) were decreased by approximately 85, 80, and 68% for Mace, Spitfire, and Volcani, respectively. In addition, a significant reduction in plant height (Figure 1E), main head length (Figure 1F), and spikelet number per head (Figure 1G) has also been observed under N stress. Flowering days (Figure 1H) were decreased by 3.2, 4.9, and 7.9%, and the GPC (Figure 1I) decreased by 4.1, 9.3, and 29.5% in Mace, Spitfire, and Volcani, respectively. A significant negative impact of low N on leaf area and peduncle length has also been noticed (Figures 1J–L). Overall, the influence of N stress on growth and agronomic traits was variable across the cultivars, where Spitfire and Volcani were more affected compared to Mace.

### Overview of RNA-Seq Transcriptome Profile in Response to Nitrogen Stress

**FIGURE 2:**
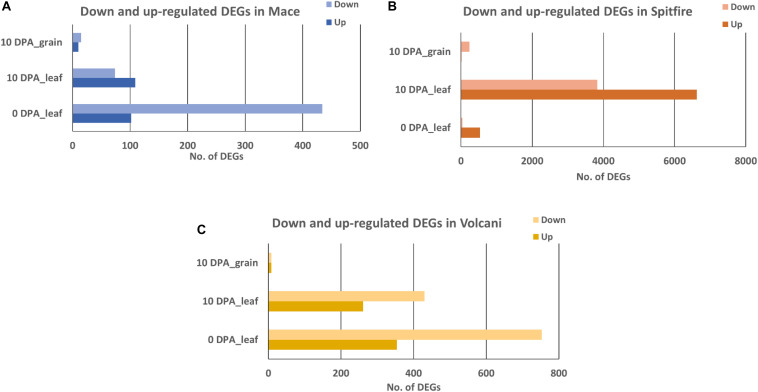
Number of up- and down-regulated expressed at 0 and 10 DPA DEGs in wheat cultivars **(A)** Mace, **(B)** Spitfire, and **(C)** Volcani. Differentially expressed genes (DEGs), 0 days post anthesis (0 DPA), 10 days post anthesis (10 DPA).

A total of 12,108 DEGs in leaf tissue and 276 DEGs in grain tissue were identified under the N stressed condition. Mace, Spitfire, and Volcani had 699, 10,535, and 1671 DEGs in second leaf and another 25, 252, and 16 DEGs in grain tissue, respectively. In the second leaf tissue, under N stress, the total up- and down-regulated DEGs at two different time points were variable across the cultivars. In Mace, at 0 DPA, the down-regulated and up-regulated DEGs were 434 and 102, respectively. At 10 DPA, the up-regulated and down-regulated DEGs were 109 and 74, respectively. Similarly, in Volcani, the down-regulated DEGs at 0 and 10 DPA were counted as 753 and 430, respectively, whereas the up-regulated DEGs were 354 at 0 DPA and 261 at 10 DPA. Cultivar Spitfire showed 536 up-regulated and 39 down-regulated DEGs at 0 DPA, whereas it showed 6624 up-regulated and 3830 down-regulated DEGs at 10 DPA. On the other hand, in grain tissue at 10 DPA, the down-regulated DEGs were 5, 237, and 8, while the up-regulated DEGs were 0, 15, and 8 identified in Mace, Spitfire, and Volcani, respectively. Variation in up- and down-regulated genes across the cultivars can be related to the difference in their response to N stress (Figure 2).

### Common DEGs Between Leaf and Grain

A total of 50 common DEGs were identified between the second leaf and grain tissue under the N stressed condition, of which 30 were down-regulated and 7 were up-regulated in both tissues. Thirteen DEGs showed inconsistent up- and down-regulation (Supplementary Table 3). Several stress-related genes have been identified among those common DEGs with >log2 fold change, including plasma membrane ATPase, Serine protease HtrA-like, transcription factor AS2/LOB, etc. Several transmembrane transport-related proteins including sulfate transporter, glycosyltransferase, and WAT1-related protein were also common in second leaf and grain tissues. On the other hand, NUE-related glutamine synthetase and glutamine dumper were significantly up-regulated in second leaf tissues but down-regulated in the grain tissue of Volcani, indicating their tissue-specific expression. Another gene related to amino acid metabolism, isoaspartyl peptidase/L-asparaginase, was up-regulated in the second leaf and grain tissue of Spitfire and Mace, indicating non-specific tissue expression. In general, the common down-regulating DEGs were largely involved in carbohydrate metabolic process (chitinase, trehalose-6-phosphate synthase) and oxidation–reduction process (aldehyde dehydrogenase, peroxidase, methyl sterol monooxygenase 1-2, gibberellin 20 oxidase 2, catalase). The up-regulating common DEGs are involved in N compound metabolic process (glutamine synthetase), sulfate transmembrane transport (sulfate transporter), and amino acid metabolism (aminotransferase like protein, isoaspartyl peptidase/L-asparaginase).

### Common DEGs Between 0 and 10 DPA

**FIGURE 3:**
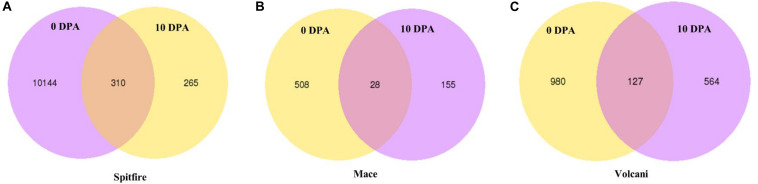
Consistently expressed DEGs at two time points in the leaf tissue of wheat cultivars **(A)** Spitfire, **(B)** Mace, and **(C)** Volcani. Differentially expressed genes (DEGs), 0 days post anthesis (0 DPA), 10 days post anthesis (10 DPA).

Under N stress, some DEGs showed consistent up- or down-regulation at both 0 and 10 DPA (Figure 3) despite the fact that they were variable between the cultivars. For example, in Mace, a total of 28 DEGs were commonly expressed at both 0 and 10 DPA, and of them, 23 were down-regulated and 5 were upregulated under N stress. Some of those DEGs showed high fold change (> + 2 or < −2) including plant protein DUF1589 of uncharacterized protein function, gibberellin receptor GID1A, catalase, a two-component response regulator, and cytochrome P450 was down-regulated, whereas RADIALIS-like TF, glycosyltransferase, and receptor-like protein kinase were up-regulated. In contrast, in Spitfire, among the 310 commonly expressed DEGs at 0 and 10 DPA, 16 showed down-regulation and 294 showed up-regulation under N stress. Among the DEGs in Spitfire, the Dof zinc finger protein, two-component response regulator, glycine-rich protein-A3, and calcium-dependent protein kinase 15 were down-regulated (log2 fold change < −4.0), and the cinnamoyl CoA reductase, receptor-like kinase, protein kinase-like, translation initiation factor IF-2, aspartate-tRNA ligase, and a beta-glucosidase were up-regulated under N stress. In Volcani, a total of 127 DEGs were found to be expressed both at 0 and 10 DPA under N stress. Of them, 86 were down-regulated and 38 were up-regulated commonly at both DPA, whereas three DEGs were down-regulated at 0 DPA but up-regulated at 10 DPA. The top down-regulated DEGs included a chlorophyll a-b binding protein, methyltransferase, endo-1,3 beta-glucanase, and plant protein DUF1589 of uncharacterized protein function, whereas the top up-regulated DEGs included cinnamoyl CoA reductase, MYB TF, glycosyltransferase, and beta-glucosidase (Supplementary Table 4).

### Common DEGs Among Cultivars

Venn diagram analysis was used to identify the number of common DEGs among the cultivars (Hulsen et al., 2008). In the second leaf tissue, down-regulated 4 DEGs at 0 and 10 DEGs at 10 DPA whereas only 3 up-regulated DEGs at 10 DPA were found in common. The common down-regulated DEGs were identified as glycine-rich protein A3, calcium-dependent protein kinase 15, etc. The common up-regulated DEGs were identified as sulfate transporter and L-allo-threonine aldolase, which is related to amino acid metabolism. However, in grain tissue, only two down-regulated DEGs identified across the three cultivars were annotated as LOB-domain containing proteins (Table 1).

**TABLE 1:** List of DEGs common among wheat cultivars: Mace, Spitfire, and Volcani.

**Tissue**	**Up-/down-regulation**	**Stage**	**Gene_id**	**Annotation**	**Log2 fold change**		
					**Volcani**	**Spitfire**	**Mace**
Leaf	Down	0 DPA	TraesCS3A02G439500LC	Glycine-rich protein A3	−5.816	−4.5188	−5.5826
			TraesCS3D02G150400LC	Glycine-rich protein A3	−5.4414	−3.2827	−4.9171
			TraesCS4A02G245300	Protein DETOXIFICATION	−6.9194	−2.5502	−4.1621
			TraesCS3D02G150300LC	Calcium-dependent protein kinase 15	−4.3632	−4.3883	−5.9832
	Down	10 DPA	TraesCS2D02G555300	ARM repeat superfamily protein	−2.7152	−3.2171	−3.3102
			TraesCS2D02G259200	Two-component response regulator	−1.9826	−2.7094	−2.2783
			TraesCS1B02G388700	Methyltransferase	−6.0313	−7.4915	−4.9876
			TraesCS6B02G051800	Glycerol-3-phosphate acyltransferase	−2.6867	−2.1339	−2.0549
			TraesCS7D02G516800	Chaperone protein dnaJ	−1.8551	−3.0128	−2.0382
			TraesCS3D02G144900	Protein DJ-1	−3.295	−5.0529	−3.237
			TraesCS5A02G472500	Amino acid transporter, putative	−4.416	−2.385	−2.4211
			TraesCS2B02G277300	Two-component response regulator	−2.7784	−3.5708	−2.8394
			TraesCS3D02G316900LC	Nucleoside triphosphatase I	−4.4166	HN	−4.1874
			TraesCS7D02G388400	Tryptophan synthase beta chain	−2.9456	−1.4694	−1.9753
	Up	10 DPA	TraesCS7D02G084100	Sulfate transporter	2.0942	3.4823	2.2596
			TraesCS7B02G128800	Epoxide hydrolase 2	4.7394	1.3931	2.5759
			TraesCS2D02G379000	L-allo-threonine aldolase	3.7648	2.4275	2.1093
Grain	Down	10 DPA	TraesCS2A02G194500	LOB domain-containing protein, putative	−1.3364	−2.2599	−1.6826
			TraesCS2D02G193400	LOB domain-containing protein	−1.5747	−2.1909	−1.8922

*Differentially expressed genes (DEGs), 0 days post anthesis (0 DPA), 10 days post anthesis (10 DPA).*

**FIGURE 4:**
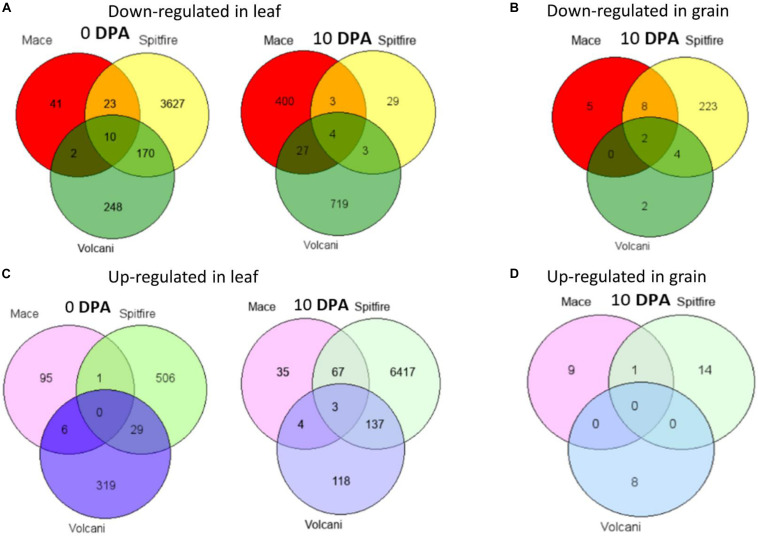
Venn diagrams of DEGs shared among Mace, Spitfire, and Volcani in leaf and grain tissues at two development stages. **(A)** The number of down-regulated genes in leaf. **(B)** The number of down-regulated genes in grain. **(C)** The number of up-regulated genes in leaf. **(D)** The number of up-regulated genes in grain. Differentially expressed genes (DEGs), 0 days post anthesis (0 DPA), 10 days post anthesis (10 DPA).

While considering the common DEGs between two cultivars, in most cases, the highest number of DEGs was common between Spitfire and Volcani among all combinations (Figure 4). The major common down-regulated DEGs between Spitfire and Volcani in second leaf were identified as methyltransferase, chlorophyll a-b binding protein, methyltransferase, and aquaporin, whereas the common up-regulated DEGs were identified as aminotransferase, early light-induced protein, F-box domain-containing protein, and glycosyltransferase. In grain tissues, among the four common down-regulated DEGs between Spitfire and Volcani, cysteine proteinase inhibitor and Ureide permease-like protein are related to N metabolism. The top down-regulated DEGs common between Mace and Volcani were identified as photosystem II 10 kDa polypeptide family proteins, chlorophyll a-b binding protein, and plant protein DUF1589 of uncharacterized protein function, whereas plasma membrane ATPase and glycosyltransferase were found as the common top up-regulated DEGs. Similarly, the common top up-regulated DEGs between Mace and Spitfire were identified as a vacuolar-processing enzyme, a boron transporter, a nuclease S1, and cytochrome P450 family protein, whereas down-regulated DEGs were identified as haloacid dehalogenase-like hydrolase (HAD) superfamily protein and thaumatin-like protein.

### Gene Ontology Reflects the Function of DEGs in Response to Nitrogen Stress

**FIGURE 5:**
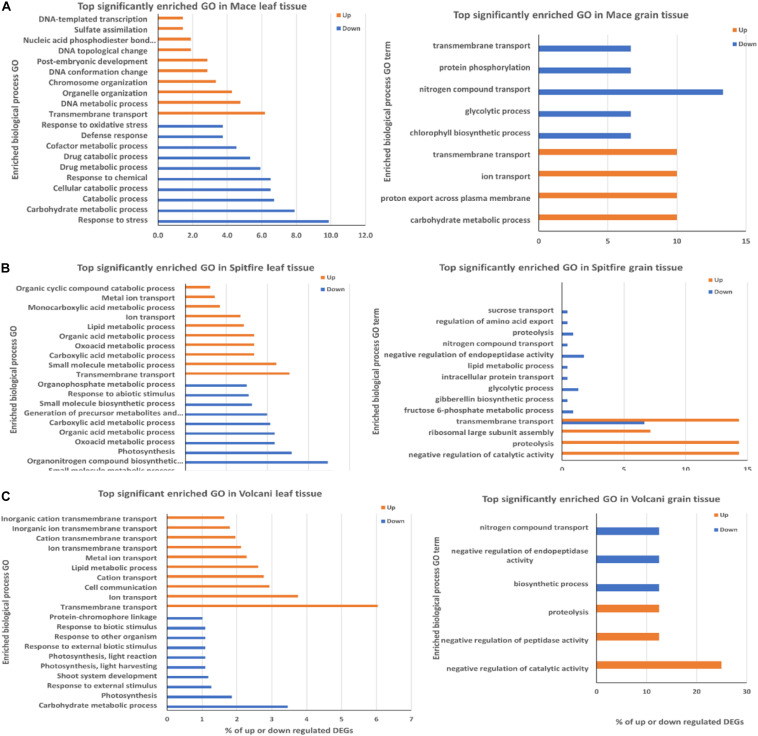
Top biological process GO terms in leaf and grain tissue of wheat cultivars **(A)** Mace, **(B)** Spitfire, and **(C)** Volcani. The frequency of the GO term is shown as percentage of the genes related to the GO term. GO, gene ontology.

The top 10 biological process GO terms characteristic to the DEGs are presented in Figure 5. The frequency of the GO term is shown as percentage of the genes compared to the total gene number related to the GO term.

In the case of the second leaf tissue, transmembrane transport GO term appeared as the top group within up-regulated DEGs in all three cultivars. Notably, another three top GO terms were common in Spitfire and Volcani, which were ion transport, lipid metabolic process, and metal ion transport, indicating that these two cultivars have some common physiological response mechanisms to N stress. In contrast, Mace did not have any other top 10 GO common with either cultivar. DNA metabolic process and organelle organization are the next top GO terms for cultivar Mace. On the other hand, genes showing decreased expression under N stress in Mace second leaf tissue were mostly involved in defense response and carbohydrate metabolic process. In Spitfire, decreasing gene expression was largely related to photosynthesis and light harvesting, organonitrogen compound biosynthesis process, and small molecule biosynthetic process. Similarly, in Volcani, genes with decreased expression patterns were also related to photosynthesis, carbohydrate metabolic process, and response to external stimulus.

In the grain tissue, the transmembrane transport process GO term was the top enriched group among the up-regulated DEGs in Mace and Spitfire. Mace also showed enrichment in carbohydrate metabolic process and ion transport. However, proteolysis and negative regulation of catalytic activity were common in Spitfire and Volcani among the top 10 enriched GO terms. Nitrogen compound transport appeared as the common GO term in all three cultivars among the down-regulated DEGs. Mace did not show enrichment for negative regulation of endopeptidase activity and proteolysis like Spitfire and Volcani. However, in Mace and Spitfire, glycolysis process was the top enriched down-regulated GO term.

### KEGG Analysis Spanned Function of DEGs in Response to Nitrogen Stress

Using the well-annotated rice genome as a reference, KEGG pathway enrichment analysis identified significantly enriched metabolic pathways and signal transduction pathways associated with DEGs. The top 20 most significantly enriched pathways were selected to produce the KEGG scatter plot (Supplementary Figure 1). Results for the KEGG pathway terms that were significant at adjusted *p*-value q-≤0.5 are shown in Tables 2, 3 for second leaf and grain, respectively. Under N stress, a total of 41 KEGG pathway terms were significantly associated with 12,108 DEGs in the second leaf, and 14 KEGG pathway terms were associated with 276 DEGs in the grain tissue. Among the 41 significant KEGG terms for the second leaf tissue, 3, 15, and 6 KEGG terms were specific to Mace, Spitfire, and Volcani, respectively, whereas one KEGG term was common between Mace and Spitfire, six KEGG terms between Mace and Volcani, and five KEGG terms between Spitfire and Volcani (Table 2). There were five KEGG terms common in all three cultivars under N stress, namely, phenylpropanoid biosynthesis, biosynthesis of secondary metabolites, flavonoid biosynthesis, metabolic pathways, and starch and sucrose metabolism. Among the 14 significant KEGG terms associated with DEGs in grain, eight, four, and one KEGG terms were specific to Mace, Spitfire, and Volcani, respectively (Table 3). The DEGs in the grain of all three cultivars were commonly associated with the KEGG pathway term glycolysis/gluconeogenesis. Among the significant KEGG terms for DEGs in the second leaf, zeatin biosynthesis, arginine and proline metabolism, and sulfur metabolism were specific to Mace, with terms like plant–pathogen interaction, photosynthesis, pentose phosphate pathway, porphyrin, and chlorophyll metabolism specific to Spitfire and beta-alanine metabolism, tryptophan metabolism, ubiquinone, and other terpenoid-quinone biosynthesis pathways found only in DEGs of Volcani. In the grain tissue, the KEGG pathways specific to Mace were glycerolipid metabolism, sphingolipid metabolism, porphyrin and chlorophyll metabolism, and galactose metabolism, whereas the pathways specific to Spitfire were alanine, aspartate and glutamate metabolism, glycine, serine, and threonine metabolism, and ribosome biogenesis in eukaryotes. The pathways specific to Volcani were cysteine and methionine metabolism. In addition, some KEGG pathways were common between two cultivars only, e.g., Mace and Spitfire had a MAPK signaling pathway common in the second leaf and glycolysis/gluconeogenesis common in the grain tissue. The KEGG terms common in Mace and Volcani included glutathione metabolism, galactose metabolism, and ABC transporters, whereas biosynthesis of amino acids, photosynthesis–antenna proteins, and circadian rhythm–plant pathways were common between Spitfire and Volcani.

**TABLE 2:** Significantly (adjusted *p*-value ≤ 0.5) enriched KEGG pathways in Mace, Spitfire, and Volcani under nitrogen stress in second leaf tissue.

**Cultivar**	**KEGG pathway**	**KEGG id**
Mace	Zeatin biosynthesis	osa00908
	Arginine and proline metabolism	osa00330
	Sulfur metabolism	osa00920
Spitfire	Plant–pathogen interaction	osa04626
	Carbon metabolism	osa01200
	Glyoxylate and dicarboxylate metabolism	osa00630
	Photosynthesis	osa00195
	Glycine, serine, and threonine metabolism	osa00260
	Carbon fixation in photosynthetic organisms	osa00710
	Glycolysis/gluconeogenesis	osa00010
	Fructose and mannose metabolism	osa00051
	Alanine, aspartate, and glutamate metabolism	osa00250
	Taurine and hypotaurine metabolism	osa00430
	Pentose phosphate pathway	osa00030
	One carbon pool by folate	osa00670
	Porphyrin and chlorophyll metabolism	osa00860
	Histidine metabolism	osa00340
	Ascorbate and aldarate metabolism	osa00053
Volcani	Beta-alanine metabolism	osa00410
	Terpenoid backbone biosynthesis	osa00900
	Ubiquinone and other terpenoid-quinone biosynthesis	osa00130
	Tryptophan metabolism	osa00380
	Butanoate metabolism	osa00400
	Phenylalanine, tyrosine, and tryptophan biosynthesis	osa00651
Mace–Spitfire	MAPK signaling pathway—plant	osa4016
Mace–Volcani	Stilbenoid, diarylheptanoid, and gingerol biosynthesis	osa00945
	Plant hormone signal transduction	osa04075
	Phenylalanine metabolism	osa00360
	Galactose metabolism	osa00052
	ABC transporters	osa02010
	Glutathione metabolism	osa00480
Spitfire–Volcani	Biosynthesis of amino acids	osa01230
	Photosynthesis-antenna proteins	osa00196
	Circadian rhythm—plant	osa04712
	Cyanoamino acid metabolism	osa00460
	Cysteine and methionine metabolism	osa00270
Mace–Spitfire–Volcani	Phenylpropanoid biosynthesis	osa00940
	Biosynthesis of secondary metabolites	osa01110
	Flavonoid biosynthesis	osa00941
	Metabolic pathways	osa01100
	Starch and sucrose metabolism	osa00500

**TABLE 3:** Significantly (adjusted *p*-value ≤ 0.5) enriched KEGG pathway in Mace, Spitfire, and Volcani under nitrogen stress in grain tissue.

**Cultivar**	**KEGG pathway**	**KEGG id**
Mace	Galactose metabolism	osa00052
	Oxidative phosphorylation	osa00190
	Glycerolipid metabolism	osa00561
	Sphingolipid metabolism	osa00600
	Glycosphingolipid biosynthesis—globo series	osa00603
	Porphyrin and chlorophyll metabolism	osa00860
	Biosynthesis of secondary metabolites	osa01110
	RNA degradation	osa03018
Spitfire	Alanine, aspartate, and glutamate metabolism	osa00250
	Alanine, aspartate, and glutamate metabolism	osa00250
	Glycine, serine, and threonine metabolism	osa00260
	Ribosome biogenesis in eukaryotes	osa03008
Volcani	Cysteine and methionine metabolism	osa00270
Mace–Spitfire	Glycolysis/gluconeogenesis	osa00010

### Protein–Protein Interaction Network Analysis of DEGs

**FIGURE 6:**
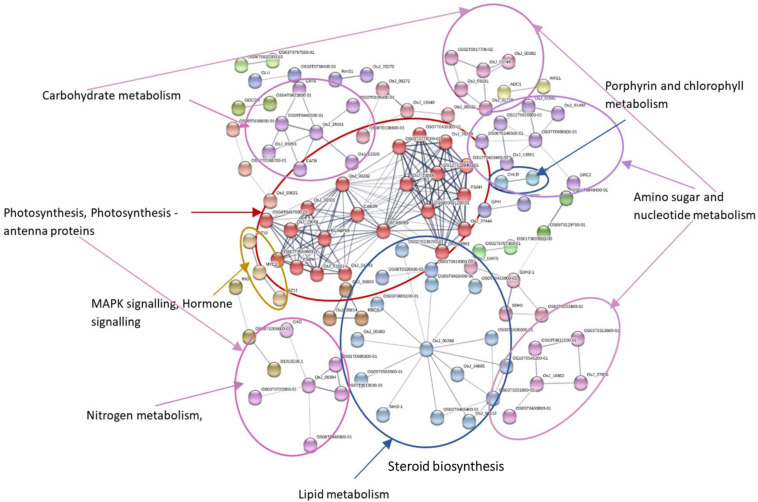
Protein–protein interaction network analysis of DEGs under N stress using *Oryza sativa* as reference. The different highlighted color indicates the different clusters of DEGs involved in different KEGG pathways. Differentially expressed genes (DEGs), Kyoto Encyclopedia of Genes and Genomes (KEGG).

**FIGURE 7:**
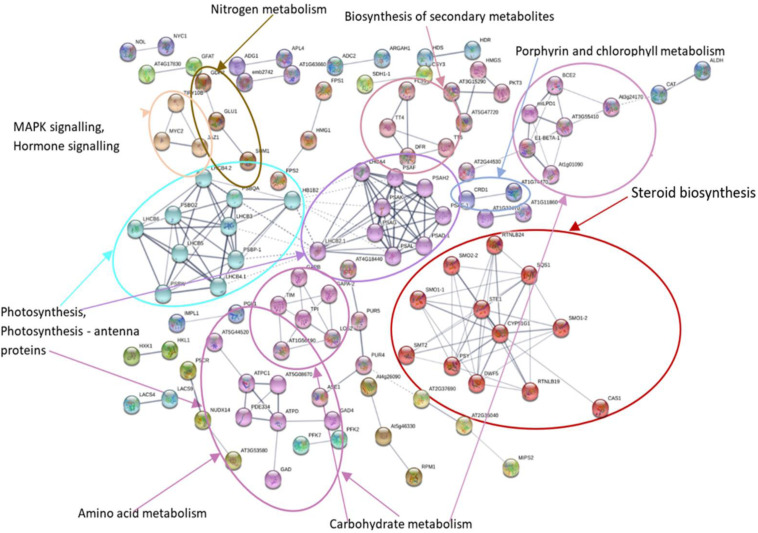
Protein–protein interaction network analysis of DEGs under N stress using *Arabidopsis thaliana* as reference. The different highlighted color indicates the different clusters of DEGs involved in different KEGG pathways. Differentially expressed genes (DEGs), Kyoto Encyclopedia of Genes and Genomes (KEGG).

MCL clustering using the inflation parameter 1.70 was used to show the association of clusters in KEGG pathways (Figures 6, 7). Networks showed that a large number of proteins were involved in multiple interactions and grouped into seven major clusters. Among the seven clusters, two large clusters were enriched in photosynthesis and steroid biosynthesis. All the interacting DEGs identified as photosynthesis-related, and photosynthesis antenna proteins were down-regulated while some DEGs related to steroid biosynthesis were up- or down-regulated. Among the other clusters, the majority of down-regulated DEGs were involved in carbohydrate metabolism, amino sugar, and nucleotide metabolism, whereas up-regulated DEGs were mostly related to amino acid metabolism and signaling. In the biosynthesis of secondary metabolites, both the up- and down-regulated DEGs were involved. Overall, the number of down-regulated DEGs was higher in the network and was mainly involved in photosynthesis and photosynthesis-antenna proteins.

### Identification of Nitrogen Metabolism-Related Genes in Response to Nitrogen Stress

N metabolism is a vital biological process in plants that determines crop productivity and yield (Stitt et al., 2002; Cai et al., 2009). The DEGs involved in N uptake, transport, and assimilation were listed separately and are presented in Table 4. Most of the N metabolism-related DEGs showed up-regulation under N stress. Among the most significant DEGs (fold change >2.0), 65% were up-regulated and 35% were down-regulated (Table 5). Spitfire showed abundancy for N metabolism-related DEGs compared to Mace and Volcani. The top up-regulated N metabolism-related DEGs included amino acid permease, glutamate dehydrogenase, low-affinity nitrate transporter protein NRT1/PTR family 1.1, tyrosine aminotransferase, and high-affinity nitrate transporter, whereas the top down-regulated DEGs included amino acid transporter family protein, nitrate transporter 1.1, nitrate transporter 1.2, nitrate reductase, and tryptophan aminotransferase. Spitfire showed the most induction ratio for protein NRT1/PTR FAMILY 1.1 (log2 fold change 6.4) and tyrosine aminotransferase (log2 fold change 5.74). Mace showed up-regulation of cationic amino acid transporter and down-regulation of amino acid transporter family proteins, amino acid permease, and protein NRT1/PTR FAMILY 1.1. Volcani showed up-regulation of amino acid permease, nitrate transporter protein NRT1/PTR FAMILY 5.5, and ammonium transporter and down-regulation of isoaspartyl peptidase/L-asparaginase, nitrate transporter 1.1 and 1.2, and tryptophan aminotransferase.

**TABLE 4:** Differentially expressed genes (DEGs) involved in nitrogen uptake, transport, and assimilation.

Name	**NUE class**	**DEG count (current study)**	**NUE related effect**	**Host**	**References**
AAT	Amino acid transporter	57	Important for early seed development	Arabidopsis	Schmidt et al., 2007
AMT	Ammonium transporter	5	Enhanced ammonium permeability improves growth and yield	Rice	Ranathunge et al., 2014
CAT	Cationic amino acid transporter	12	Involved in intracellular amino acid storage and mobilization	Arabidopsis	Yang H. et al., 2015
CLC	Chloride channel protein	14	Enhanced N assimilation and tolerance to stress	Oilseed rape	Liao et al., 2018
LHT	Lysine/histidine transporter	1	Disruption of LHT lead to growth inhibition and low yield	Rice	Wang X. et al., 2019
NRT	Nitrate transporter	36	Suppression of NO${}_{3}^{-}$-starvation-induced leaf senescence	Arabidopsis	Meng et al., 2016
OPT	Oligopeptide transporter	5	Essential for embryo development	Arabidopsis	Stacey et al., 2002
AGT	Alanine: glyoxylate aminotransferase	2	Catalyze transamination reaction in peroxisome	Arabidopsis	Liepman and Olsen, 2001
ASN	Asparagine synthetase	1	Regulation of plant development and tiller outgrowth	Rice	Lu et al., 2018
AspAT	Aspartate aminotransferase	5	Overexpression related to increase amino acid content in seed	Rice	Zhou et al., 2009
GDH	Glutamate dehydrogenase	3	Played important role in nitrogen metabolism and plant growth, and grain yield	Rice	Abiko et al., 2010
GOGAT	Glutamate synthase	3	Increased ammonium assimilation in root	Arabidopsis	Konishi et al., 2014
GS	Glutamine synthetase	7	Knockdown negatively affect plant growth, spikelet no., grain weight	Rice	Tabuchi et al., 2005
NR	Nitrate reductase	2	Increase lateral root formation under partial nitrate nutrition	Rice	Sun et al., 2015
NiR	Nitrite reductase	3	Increased nitrite assimilation	Arabidopsis	Takahashi et al., 2001
TS	Threonine synthase	3	Inhibition related to high methionine biosynthesis	Potato	Zeh et al., 2001
TrP	Tryptophan aminotransferase	3	Improved grain yield	Wheat	Shao et al., 2017
TAT	Tyrosine aminotransferase	1	Differentially expressed between low and high nitrogen treatments	Wheat	Current study
GDU	Glutamine dumper	2	Involved in export of amino acids	Arabidopsis	Pilot et al., 2004

**TABLE 5:** Up- and down-regulated nitrogen metabolism-related DEGs identified under nitrogen stress in three wheat cultivars (Mace, Spitfire, and Volcani).

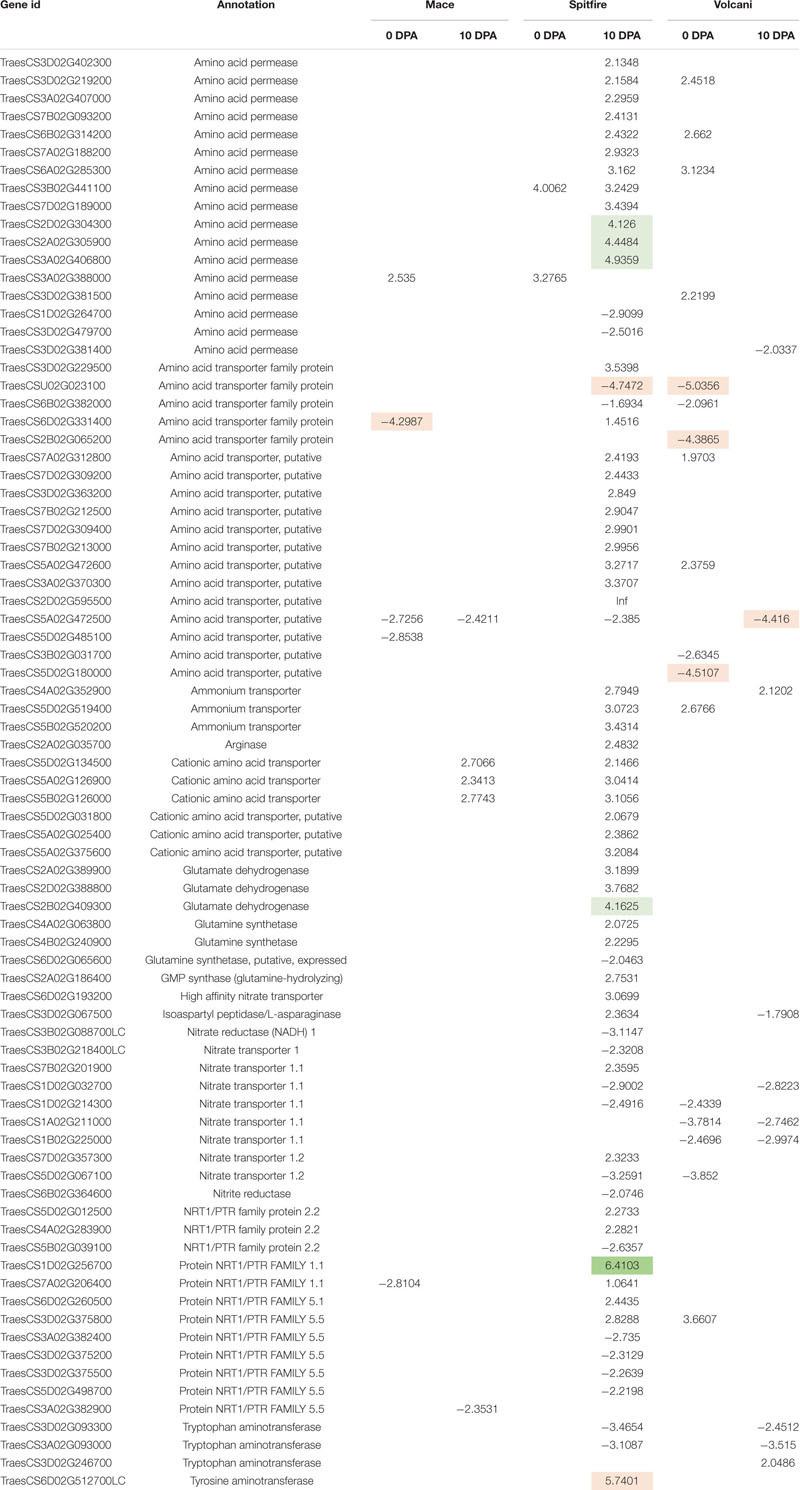

*The green highlighted values stand for expressional change strength when log2 fold change >2.0 whereas brown when log2 fold change < −2.0. The intensity of color increases with the increase in degree strength. Inf is an indication of differential up-regulation strength when the respective gene only expressed at low N condition. Differentially expressed genes (DEGs), 0 days post anthesis (0 DPA), 10 days post anthesis (10 DPA).*

### Identification of Common Nitrogen Stress-Responsive Genes Across the Cultivars

Identification of the common DEGs between two N treatments included genes from 6 pair comparisons (2 developmental stages × 3 cultivars) for leaf and 3 pair comparisons (1 developmental stage × 3 cultivars) for grain tissue. In the second leaf, a total of 14 up-regulated and 42 down-regulated DEGs were identified that were common in all three cultivars (Tables 6, 7). Among the 14 up-regulated common DEGs, aldo/keto reductase family protein, nuclease S1, alcohol dehydrogenase, putative, placenta-specific8 (PLAC8) family protein, and sulfate transporter showed relatively high (log2) fold change. Eight of the 42 down-regulated DEGs in the leaf tissue showed high log2 fold change, including 3 photosystem II 10 kDa polypeptide family protein, 2 methyltransferases, chlorophyll a-b binding protein, cytoplasmic dynein 2 heavy chain 1, and plant protein 1589 of uncharacterized protein function. However, the only two down-regulated DEGs were commonly expressed in the grain tissue involved LOB domain-containing proteins. These common genes can be considered important N responsive genes.

**TABLE 6:** Top common up-regulated DEGs identified among wheat cultivars Mace, Spitfire, and Volcani under nitrogen stress.

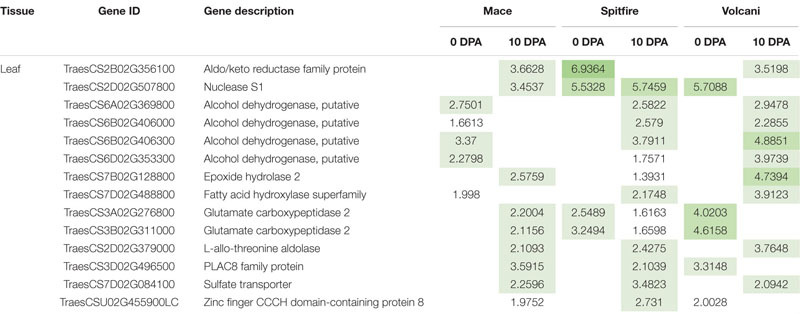	

*The green highlighted values stand for expressional change in strength when log2 fold change >2.0. The intensity of color increases with an increase in the degree of strength. No common up-regulated DEGs among the three cultivars were found in grain tissue. Differentially expressed genes (DEGs), 0 days post anthesis (0 DPA), 10 days post anthesis (10 DPA).*

**TABLE 7:** Top common down-regulated DEGs identified among wheat cultivars Mace, Spitfire, and Volcani under nitrogen stress.

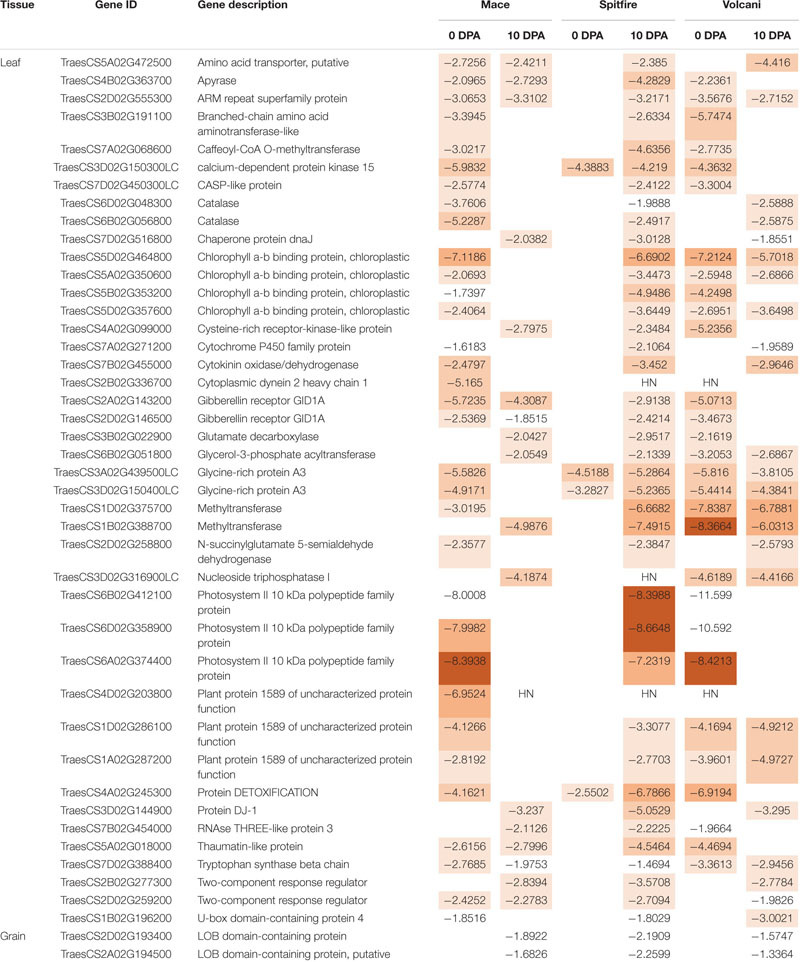

*The brown highlighted values stand for expressional change in strength when log2 fold change < −2.0. The intensity of color increases with an increase in the degree of strength. Differentially expressed genes (DEGs), 0 days post anthesis (0 DPA), 10 days post anthesis (10 DPA).*

To reveal the high N responsive genes, the top 10 up-regulated and top 10 down-regulated DEGs were selected in three cultivars. The log2 fold change value of each group is shown in Supplementary Tables 5, 6 for the second leaf and Supplementary Tables 7, 8 for grain. In the second leaf tissue, the stress-associated glutathione S-transferase (GST), RADIALIS-like TF, and plasma membrane ATPase were the most N responsive up-regulated DEGs in Mace. In Spitfire, the top N responsive up-regulated DEGs were isocitrate lyase, laccase, and 11S globulin seed storage protein 2 related to carbon metabolism, lignin metabolism, and nutrient reservoir, respectively, whereas in Volcani, 1-phosphatidylinositol-3-phosphate 5-kinase, caleosin, protein transport protein Sec61 subunit gamma, and elongation factor G have appeared on top. In the grain tissue, the top up-regulated DEGs in Mace were isoaspartyl peptidase/L-asparaginase, plasma membrane ATPase, and trypsin family protein. The up-regulated DEGs in Spitfire showing high responsiveness to N stress were mainly N metabolism-related and aminotransferase like protein and aspartic proteinase nepenthesin, whereas in Volcani, invertase/pectin methyl esterase inhibitor family protein, cysteine proteinase inhibitor, and glycosyltransferase that is mainly associated with proteolysis and negative regulation of proton export across plasma membrane were found more prominent. There was a prevalence of defense-related down-regulated DEGs detected in the second leaf tissue of Mace, whereas photosynthesis-related DEGs were abundant in both Spitfire and Volcani. In the grain tissue of Mace, Spitfire, and Volcani, the down-regulated DEGs were predominantly related to proteolysis and N metabolism.

**FIGURE 8:**
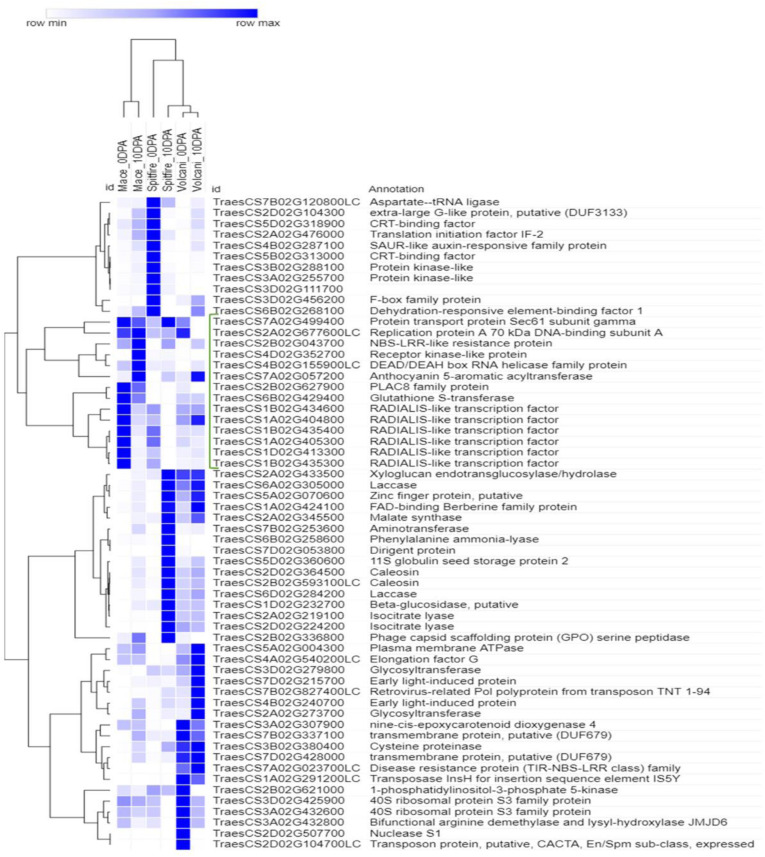
Hierarchical clustering of top up-regulated DEGs in leaf tissue. The green bracketed genes are the highly expressed up-regulated genes in high NUE wheat cultivar Mace. Differentially expressed genes (DEGs), 0 days post anthesis (0 DPA), 10 days post anthesis (10 DPA).

**FIGURE 9:**
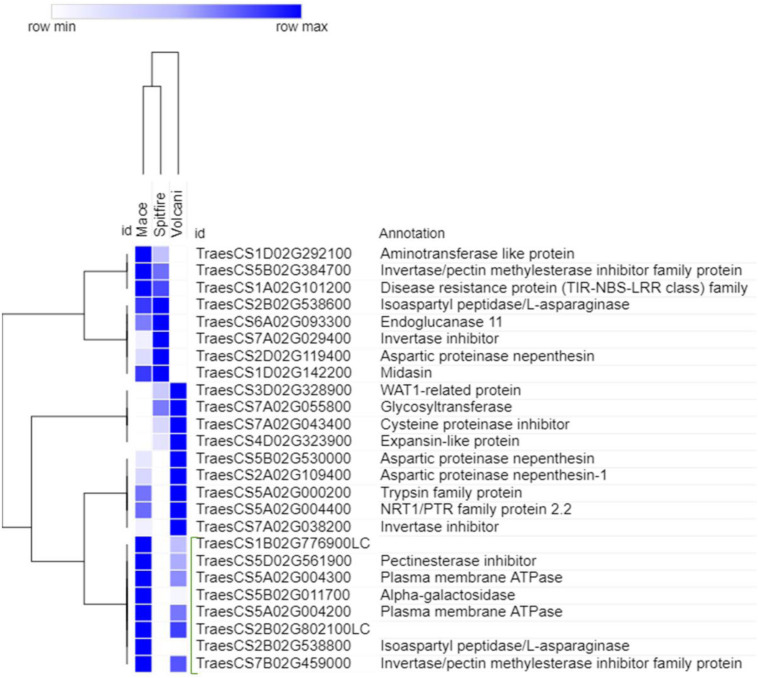
Hierarchical clustering of top up-regulated DEGs in grain tissue. The green bracketed genes are the highly expressed down-regulated genes in high NUE wheat cultivar Mace. Differentially expressed genes (DEGs).

To select the genes that can be related to the tolerance to N starvation in high NUE cultivars, further analysis was done for the top genes using hierarchical clustering (see footnote 6). The top genes that showed high read count at low N in a high NUE cultivar (Mace) can be related to its tolerance to N stress. In the second leaf tissue, the top up-regulated DEGs with high abundance were principally identified as RADIALIS-like TFs, GST, and PLAC8 family protein (Figure 8). Similarly, in the grain tissue, plasma membrane ATPase, isoaspartyl peptidase/L-asparaginase, and alpha-galactosidase were identified as the top up-regulated DEGs expressed abundantly in high NUE cultivar Mace (Figure 9).

## Discussion

To improve NUE, it is important to understand the plant response to N treatments, especially to N limitation at both physiological and transcriptome levels. Targeting improved GPC and GY, the present study aimed to explore the transcriptome response of wheat to long-term N stress and identify potential candidate genes that are differentially expressed with high relative abundance across different genotypes in common. According to previous study, the GY of Mace is higher than those of Spitfire and Volcani, whereas the GPC of Mace is relatively lower than those of Spitfire and Volcani. It was also reported that the GY and GPC of Spitfire are affected more negatively under N-limiting conditions (Alhabbar et al., 2018a). Thus, it is important to unravel the underlying genes that can contribute to N stress tolerance for further genetic manipulation study.

Inadequate supply of N negatively affects plant morphology, limits growth, and decreases biomass in wheat (van der Werf et al., 1993). Most plants exhibit prominent changes in their growth and development under N-stressed conditions. Previous studies reported that adaptations of plants with nutrient-stressed conditions are mainly dependent on morphological changes (Wang J. et al., 2019; Zhao et al., 2005). The results of this study also confirmed that low N stress inhibited wheat growth, with significant negative impact on different phenotypes (Figure 1). These results were consistent with the N stress studies in wheat (Curci et al., 2017), sorghum (Gelli et al., 2014), corn (Jin et al., 2015), and rice (Sinha et al., 2018). In Mace, GY and GPC were less affected by N stress compared to those in Spitfire and Volcani in glasshouse conditions, although under high N conditions, the GPC of Spitfire and Volcani was higher than that of Mace. In agreement with the previous study (Alhabbar et al., 2018b), this study further confirms that Mace is more tolerant of N stress. It was reported that the GY in maize was decreased by 38% with the change in N treatment from high to low (Gallais and Hirel, 2004), which can be associated with the interrupted synthesis of chlorophyll and photosynthesis performance (McCullough et al., 1994). Many studies also reported the influence of hormones and N metabolism- and nutrient stress-related genes on agronomic traits (Singh et al., 1973; Cai et al., 2009). Thus, it is predicted that under a N stress condition, many genes involved in different biological pathways are cross-talking in mitigating the adverse effect of stress instead of a single factor. However, the GPC and the number of days to flower were less affected by N stress, which explains that these parameters can be rather controlled by genotype.

Under N stress, the genes that expressed differentially were mostly leaf specific compared to grain. Also, the DEGs in the leaf were related to versatile functions, whereas a significant percentage of DEGs in the grain were related to transport and N metabolism. The 50 common DEGs between the second leaf and grain identified were mostly related to defense, amino acid metabolism, N metabolism, carbohydrate metabolism, and sulfate transport. It is known that in the plastid of the leaf, sulfate is converted to sulfide using the reducing power of photosynthesis and incorporated into amino acids that later remobilize to developing seeds (Gallardo et al., 2014; Jobe et al., 2019). Developing seeds requires sulfur amino acids to synthesize storage protein to secure germination for the next generation (Leustek et al., 2000; Saito, 2000). DEG analysis also showed a higher number of DEGs in Spitfire (10,535 in the second leaf and 252 in grain) in comparison to Volcani (1671 in the second leaf and 16 in grain) and Mace (699 in the second leaf and 25 in grain), which indicates that under N stress, Spitfire responds more actively, and that involves more signaling pathways than Volcani and Mace. Spitfire responded to N stress mostly by up-regulating, whereas Mace and Volcani responded by down-regulating the DEGs.

Precedence of any biological processes at a particular developmental stage is correlated with the changes in the expression pattern of corresponding genes involved. GO enrichment analysis is an effective method to understand the key biological processes participating in adapting stress. For instance, a N starvation study in durum wheat reported N compound metabolism, carbon metabolism, and photosynthesis as the top enriched biological processes (Curci et al., 2017). The oxidation–reduction process and metabolic process were top enriched biological processes in wheat seedlings in response to N limitation (Wang J. et al., 2019). The top enriched biological processes in rice have been reported to be associated with metabolic processes, cellular processes, and transport under N-starved conditions (Yang S. Y. et al., 2015). This study showed that the up-regulated DEGs were mainly associated with transmembrane transport, whereas the down-regulated DEGs were mainly associated with metabolic process and stress response, which supports that during grain filling, the plant increases its overall remobilization through protein degradation and transport (McCullough et al., 1994; Masclaux-Daubresse et al., 2008). Significant up-regulation of transmembrane transport, nitrogenous compound transport, and proteolysis was common in all three cultivars (Figure 5). However, in Mace, a greater percentage of up-regulated DEGs were related to DNA conformation change and sulfate assimilation, whereas in Spitfire and Volcani, DEGs were highly significant in the lipid metabolic process. The up-regulation of DNA metabolic process in Mace can be related to epigenetic change, which underlies its stability under N stress conditions. A long-term primed state of the epigenetic mechanism involves DNA conformation change such as change in chromatin structure, variation in composition and position of the nucleosome, and post-transcriptional modification to cope more efficiently with the subsequent stress (Chinnusamy and Zhu, 2009). Also, the increase in sulfate assimilation in Mace can be related to the synthesis of proteins rich in S-containing amino acids such as glutathione, which is a major component of the stress response (Yamaguchi et al., 1999; Kopriva and Rennenberg, 2004). In Spitfire and Volcani, the increase in lipid metabolism can be related to senescence (Xiao and Chye, 2011). It was reported that, during senescence, synthesis of phytyl-ester synthase is induced, which is associated with the synthesis of triglycerol and phytyl-esters of plastid fatty acids (Xiao and Chye, 2011; Troncoso-Ponce et al., 2013). In contrast to the up-regulated DEGs, a higher percentage of down-regulated DEGs in Mace were significantly related to the cellular catabolic process, which is known to be related to plant biotic and abiotic stress response (Tavladoraki et al., 2012), whereas in Spitfire and Volcani, the DEGs were more abundant in photosynthesis. The significantly decreased expression of photosynthesis-related DEGs can be related to decreased grain weight per plant in Spitfire and Volcani (Zhao et al., 2005; Boussadia et al., 2010). However, in all three cultivars, the down-regulated DEGs were more prominent in the carbohydrate metabolic process, which indicated that, regardless of genotypes, N stress can negatively affect plant carbohydrate metabolism (Rufty et al., 1988) and plants adapted to N stress by down-regulating the expression of many genes of this kind.

Kyoto Encyclopedia of Genes and Genomes analysis results also revealed that in all three cultivars, DEGs were involved in phenylpropanoid biosynthesis, biosynthesis of secondary metabolites, flavonoid biosynthesis, and sucrose and starch metabolism. The regulation of these genes in stress adaptation has been reported in several studies (Dixon and Paiva, 1995; Huang et al., 2010; Akula and Ravishankar, 2011; Petrussa et al., 2013). However, some cultivar-specific differences highlighted the importance of genetic variability in stress response (Tables 2, 3). For example, DEGs were more abundantly related to MAPK signaling in Mace and Spitfire, plant hormone signal transduction, glutathione metabolism in Mace and Volcani, photosynthesis-antenna proteins, and circadian rhythm in Spitfire and Volcani. Also, some DEGs that were significantly abundant in pathways related to zeatin biosynthesis in Mace, terpenoid biosynthesis in Volcani, and plant–pathogen interaction in Spitfire are important to identify the underlying genes related to biological pathways to develop stress-tolerant cultivars (Cheong et al., 2002; Vickers et al., 2009).

**FIGURE 10:**
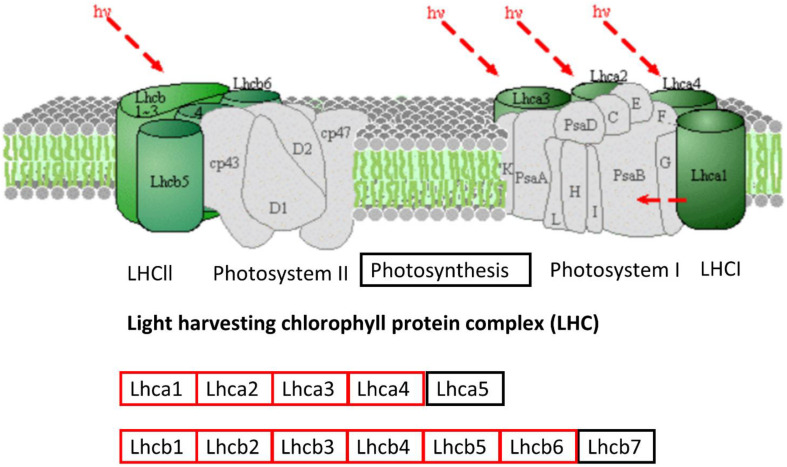
Down-regulated protein orthologous (*Arabidopsis thaliana*) of differentially expressed genes in photosynthesis-antenna proteins pathways. The proteins in red are down-regulated. The pathway map was generated using KEGG (Kanehisa et al., 2016, 2017).

Protein–protein interaction analysis has been used to identify DEGs that are interacting in different biological processes such as photosynthesis, photosynthesis-antenna proteins, and steroid biosynthesis. Photosynthesis is the vital biological process by which plants absorb light energy and assimilate CO_2_ to produce dry matter and comprises reactions that are regulated by proteins in the chloroplast (Chandna and Ahmad, 2015). Within this highly interactive and regulated system, change in one component can cause changes to other components. The strength of photosynthesis capacity is mainly dependent on the N content of chloroplasts in the leaf (Evans, 1989; Evans and Poorter, 2001; Ripullone et al., 2003). Numerous studies have reported that N significantly affects photosynthesis (Wei et al., 2016; Lin et al., 2017) through its association with the light reaction in the chloroplast and/or the dark reaction (Sage et al., 1988; Von Caemmerer, 2000). The light-harvesting complex (LHC) comprises chlorophylls a and b and the chlorophyll a-b binding protein and is closely associated with photosystem I and II. LHC plays an important role as a light receptor that captures and delivers the excitation energy between two photosystems and adjusts the distribution of excitation energy by being phosphorylated reversely under changing light conditions (Sage et al., 1988). The PSII outer antenna LHCB proteins are important components of the major LHC, and they consist of minor antenna complexes LHCB4 (CP29), LHCB5 (CP26), and LHCB6 (CP24) and major antenna complexes that comprise homo- and heterotrimers of LHCB1, LHCB2, and LHCB3 (Jansson, 1994, 1999). In the present study, all the chlorophyll a-b binding proteins that interacted with each other in adjusting N stress were down-regulated. In agreement with the study in rice seedlings in a water-stressed environment (Dalal and Tripathy, 2018), the current study identified significantly decreased expression of components of LHCs of both PSII and PSI (Figure 10 and Supplementary Table 9). Moreover, the decreased photosynthesis rate and chlorophyll content under N-stressed condition (Figure 1) can be related to the differential expression of chlorophyll a-b binding proteins.

**FIGURE 11:**
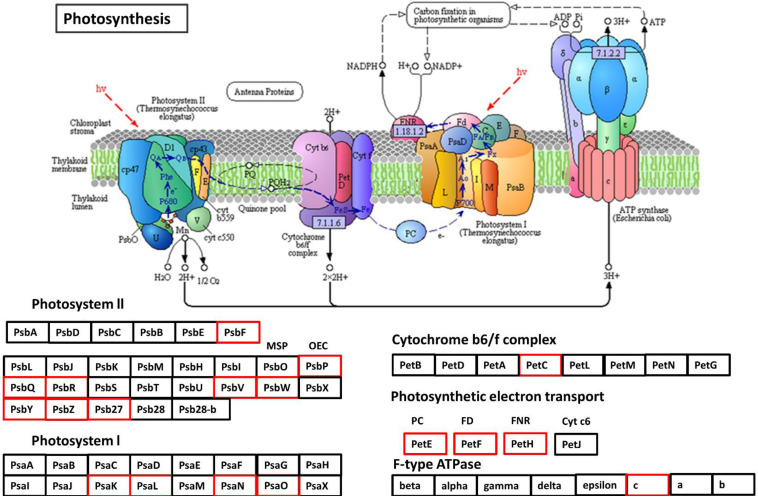
Down-regulated protein orthologous (*Arabidopsis thaliana*) of differentially expressed genes in photosynthesis pathways. The proteins in red are down-regulated. The pathway map was generated using KEGG (Kanehisa et al., 2016, 2017).

The rate of photosynthesis has an intense positive correlation with N status in soil (Makino et al., 2003; Nunes-Nesi et al., 2010). Under N stress, a plant might adapt by reduced chloroplast surface area and a decreased light energy absorption, which can affect photosynthesis negatively (Li et al., 2009, 2013; Muller et al., 2009; Georgieva et al., 2010). In the present study, many PSII and PSI subunits showed a decreased expression in low NUE cultivars Spitfire and Volcani under N-stressed condition (Figure 11 and Supplementary Table 10), which can impede photosystem repair and photosynthetic electron transport chain function (Foyer and Shigeoka, 2011). Also, the expression of cyt559 had decreased, which binds most of the cofactors in the photocatalytic activity of photosystem II. Among the down-regulated DEGs of PSII components, the core components PsbO, PsbP, and PsbQ are known to be involved in the water oxidation and its optimization process (Bricker et al., 2012). PsaK is associated with the LHCI antenna system, and PsaO plays a role in the formation of the docking site for LHCII binding to PSI (Jensen et al., 2007). The down-regulated PetC provides resistance to photo-oxidative damages by contributing to the thermal intemperance of light energy and lumenal acidification and mediates electron transfer between PSII and PSI (Munekage et al., 2001). The photosynthetic electron transport component showed down-regulation for PetE that participates in electron transfer between P700 and the cytochrome b6-f complex in photosystem I and PetF (ferredoxins are iron–sulfur proteins) transfer electrons in a wide variety of metabolic reactions (Achard et al., 2008). PetH plays a significant role in balancing cyclic and noncyclic electron flow to supply the ATP and reducing power required by the plant (Claeys et al., 2012). Moreover, F-type ATPase gamma and a c subunit aid electron transport in both photosystems I and II were also significantly down-regulated. Significantly down-regulated DEGs in low NUE cultivars Spitfire and Volcani were found as the components of the LHC system and PSI and PSII, underlying their molecular basis of low GY mechanisms. Therefore, understanding N stress-responsive DEGs that participated in photosynthesis might provide a base to improve the photoprotection capacity to sustain photosynthesis as well as improving plant N-stress tolerance.

**FIGURE 12:**
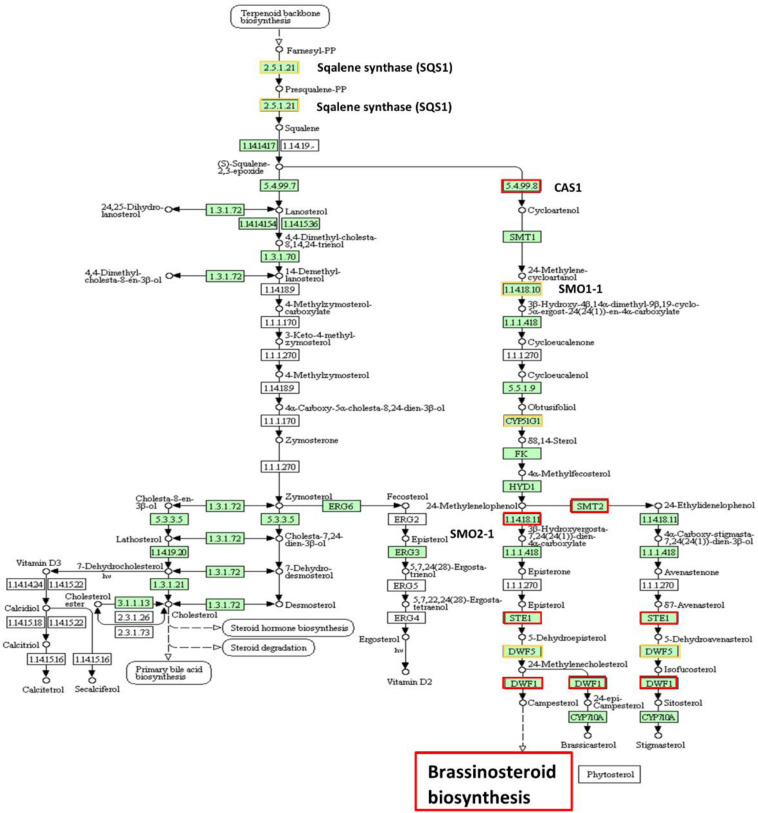
Up- and down-regulated protein orthologous (*Arabidopsis thaliana*) of differentially expressed genes in steroid biosynthesis. The protein names bordered in yellow are up-regulated and red are down-regulated. The pathway map was generated using KEGG (Kanehisa et al., 2016, 2017).

The up- and down-regulated DEGs in relation to steroid hormone biosynthesis, specifically the BR biosynthesis, in low NUE cultivars also lead to understanding the role of this hormone in N stress adaptation (Figure 12 and Supplementary Table 11). Down-regulated DEGs were more prominent compared to up-regulated DEGs in this pathway, which indicates the declined BR hormone biosynthesis. Previous studies showed exogenous application of BR enhanced photosynthesis under stress conditions (Niu et al., 2016; Shu et al., 2016). Chlorophyll is an important parameter and is commonly used to measure photosynthetic activity. However, chlorophyll is highly sensitive and responds to stress by decreasing the chlorophyll a, b content in leaves (Rehman et al., 2016). In low NUE cultivar Spitfire and medium NUE cultivar Volcani, significantly decreased level of chlorophyll a-b binding protein can be associated with their reduced chlorophyll content compared to high NUE cultivar Mace (Figure 1). Previous studies also reported that BR can reducing the harmful effect of stress by activating the synthesis of antioxidants like glutathione reductase, catalase, peroxidase, etc., contributing to increase in yield and yield components (Hayat et al., 2000; Vardhini and Anjum, 2015; Anwar et al., 2018). In high NUE cultivar Mace, the absence of significant DEGs related to BR biosynthesis that are interacted at the protein level can be related to its increased tolerance to N stress, relatively high chlorophyll content, tiller number, and grain weight per plant (Figure 1). So far, no previous study has been reported on the putative role of BRs in wheat under N-stressed conditions. Thus, identifying the involvement of BR biosynthesis provides a suitable platform to explore the essential role of BR in N stress tolerance and further application of BRs to improve wheat production.

Through annotation of the transcriptome, several known and putatively N-metabolism-related genes were identified both to be up- and down-regulated. Usually, N stress increases the expression of high-affinity transport systems for nitrate and ammonium (Crawford and Glass, 1998). Previous reports showed that high-affinity nitrate transporters were expressed in N-starved seedlings of Arabidopsis (Wang R. et al., 2003). In rice, the nitrate transporter (*OsNRT2.2*) in association with *OsNAR2.1* transports nitrate, which can promote the elongation of lateral roots (Feng et al., 2011; Li et al., 2007). In the current study, the expression of high-affinity nitrate transporters (NRT1/PTR family protein 2.2) was up-regulated under N stress. This indicates a more efficient N uptake under N-limited condition. On the other hand, the expression of most of the dual affinity nitrate transporters (like nitrate transporter 1.1) was decreased under N stress, which is known to regulate root and shoot growth (Mounier et al., 2014). The down-regulation of Protein NRT1/PTR FAMILY 5.5 and tryptophan aminotransferase can be related to the retarded growth and low GPC and GY of low N-treated plants (Won et al., 2011; Léran et al., 2015). Also, a decreased expression of nitrite reductase was observed, which is related to nitric oxide (NO) homeostasis (Chamizo-Ampudia et al., 2017). NO can act as a signaling molecule in plant immune response, defense-related gene expression, and the hypersensitive response mechanism (Mur et al., 2012). Conversely, most of the N metabolism-related DEGs of the known and putative amino acid permease, amino acid transporter, ammonium transporter, glutamate dehydrogenase, glutamine synthase family and tyrosine aminotransferase, and tryptophan aminotransferase were up-regulated. Glutamine synthetase is a key enzyme (Cai et al., 2009) that catalyzes the conversion of glutamate (Glu) to glutamine (Gln). GOGAT is involved in the transfer of the amide group of Gln to a-ketoglutarate (2-OG) to subsequently produce Glu (Cren and Hirel, 1999). Gln is involved in the biosynthesis of organic nitrogenous compounds, such as amino acids, nucleotides, and chlorophyll, and plays a major role in regulating plant N assimilation in grain production (Martin et al., 2006; Gadaleta et al., 2011). In this study, most of the significant DEGs related to N metabolism were found in Spitfire, and the lowest number of DEGs with a significant change in expression was identified in Mace. The high abundance of a nitrate transporter and ammonium assimilatory gene abundance in low NUE cultivars Spitfire and Volcani can be related to adapt the N-stressed condition. Similar results were observed in a transcriptome study with sorghum, where N assimilator genes were abundant in sensitive and low NUE cultivars (Singh et al., 1973). The smaller number of N metabolism-related DEGs can be related to a better tolerance of Mace to low N conditions. These findings are also supported by similar outcomes in rice (Lian et al., 2006).

The common DEGs that were simultaneously induced or repressed under N stress across the three cultivars are also potentially important for N stress response. Among the common down-regulated DEGs (Table 7), photosystem II 10 kDa polypeptide family protein and chlorophyll a-b binding protein are related to photosynthesis and light harvesting, which are sensitive to stress (Rehman et al., 2016; Nowicka et al., 2018). The chlorophyll content was significantly decreased under the N-stressed condition in all three cultivars compared to high N. Down-regulation of stress-responsive DEGs like catalase, thaumatin-like protein, and cytochrome P450 family protein is also known to be related to stress adaptation (Cai et al., 2013; Alam and Ghosh, 2018). Also, the expression of phytohormone-related DEGs such as gibberellin receptor GID1A and cytokinin oxidase/dehydrogenase showed down-regulation common in all three cultivars. Reduced GA levels and signaling are known to be associated with restrained growth and development of plant by inducing accumulation of DELLA (Colebrook et al., 2014), known as positive regulators of N stress-induced anthocyanin accumulation (Zhang et al., 2017). Under salt stress, the DELLA mutant has been reported to be strongly correlated with plant growth, height, time to flowering, and stress tolerance (Achard et al., 2008). Other studies also showed that GA-induced DELLA has a positive effect on stress tolerance (Claeys et al., 2012). In this study, the reduced plant height and growth can be related to a reduction in GA. Other phytohormone cytokinins (CKs) can regulate plant developmental processes under stressed conditions (Rubio-Wilhelmi et al., 2011). Recent studies reported that CKs act as a long-distance messenger that signals the N status of the plant in regulating the nutrient uptake system (Rubio-Wilhelmi et al., 2011). Overexpression of CK degradation enzyme-CKX has been known to exhibit an increased drought and salinity tolerance (Schmülling et al., 2003; Nishiyama et al., 2012). In addition, cytokinin and gibberellin also influence photosynthesis under stressed conditions (Caers et al., 1985; Biemelt et al., 2004). Some other common down-regulated genes involved glycine-rich protein A3, which binds and stabilizes the stress-inducible transcripts (Sahi et al., 2007), methyltransferase related to epigenetic tolerance to stress through DNA methylation (Boyko and Kovalchuk, 2008), calcium-dependent protein kinase 15, which functions in long-term adaptive processes or plant development by facilitating cross-talk between different Ca^2+^-mediated stress signaling pathways (Lee and Rudd, 2002; Schulz et al., 2013), and a two-component response regulator that plays a role in stress response by transducing extracellular signals to the cytoplasm through phosphotransfer between the two components (Urao et al., 1998). Amino acid metabolism-related genes like putative amino acid transporters and branched-chain amino acid aminotransferase-like proteins are common in the three cultivars and can also contribute to stress tolerance by down-regulating their expression (Good et al., 2007). Interestingly, three DEGs annotated as plant protein 1589 with uncharacterized function were all down-regulated, which are potentially important candidates for further study. The down-regulation of LOB domain-containing proteins that were common in the grain of the three cultivars was reported to be involved in lateral root formation (Liu et al., 2005; Yang W. et al., 2015). LOB domain-containing proteins are also known to control the BR hormone negatively in N metabolism as well as plant growth and development (Bell et al., 2012; Ma et al., 2017).

Some genes common in the second leaf of the three cultivars showed up-regulation (Table 6) that can facilitate tolerance to N stress to survive. Among the 14 up-regulated DEGs, 4 were annotated as putative alcohol dehydrogenase family proteins that were also reported to accumulate at an increased level under low-temperature stress in maize and rice (Christie et al., 1991). Two glutamate carboxypeptidase 2 were up-regulated that are known to negatively regulate drought and freezing stress and play a role in carbon and amino acid metabolism (Shi et al., 2013). Another up-regulated DEG annotated as PLAC8 family protein was reported to be involved in cadmium tolerance and accumulation, which can also be a good candidate to increase N stress tolerance (Wang F. et al., 2019). Also, a sulfate transporter was found to be up-regulated and has been previously reported as affected by N deficiency (Yu et al., 2018). As sulfur assimilation is important for the biosynthesis of S-containing amino acids that remobilize to develop seeds for storage protein synthesis, a sulfate transporter is worth further study under the context of NUE.

The DEGs that demonstrated a high expressional change due to N stress can be an important candidate for N stress response. Analyzing the expressional variation of genes across the cultivars with different NUE, this study proposed that the highly up-regulated genes expressed in high NUE cultivar Mace with high abundance can contribute to N stress tolerance. In the second leaf of Mace, the top abundantly expressed up-regulated DEGs (Supplementary Table 9) in Mace involved RADIALIS-like (RADL) TFs, GST, and PLAC8 family protein. GST was reported to catalyze the glutathione-dependent detoxification reactions and the reduction of hydroperoxides. It also plays a role in protection against environmental stresses by binding and sequestrating secondary metabolites like flavonoids and phenolics (Tahkokorpi et al., 2007). In maize, *ZmGSTU1* can protect plant cells from oxidative stress damage through binding and conjugating porphyrinogens. Under stress conditions and during senescence, porphyrinogens leak from chloroplast to the cytosol and become oxidized to the lipophilic and phytotoxic protoporphyrin (Dixon et al., 2010; Lederer and Böger, 2005). Binding of GST to leaked porphyrinogens can prevent their auto-oxidation, protecting plant cells from oxidative stress (Lederer and Böger, 2003). RADL TFs are a subfamily of MYB-related genes containing a single SANT (*SWI3/ADA2/N-CoR/TFIIIB*)/MYB DNA-binding domain, which is highly homologous to the RADIALIS gene product of *Antirrhinum majus*. The Antirrhinum RADIALIS gene is involved in the regulation of floral asymmetry, and mutation of this gene results in a symmetrical (or radial) floral morphology (Baxter et al., 2007). In Arabidopsis, a RADL TF (*RSM1*) is implicated in controlling early photomorphogenesis (Hamaguchi et al., 2008). In rice, overexpression of RADL3 TF (*OsRL3*) exhibits a stay-green phenotype during dark-induced senescence in an ABA-dependent pathway (Park et al., 2018). A study in lady’s bedstraw (*Galium verum*) to understand the genetic basis of morphological difference of its two variants showed that two short insertions in the promoter region of RADL1 in one variant can be related with its nonfunctionality and dwarfism (Jeong et al., 2014). In Barley, the effect of CENTRORADIALIS (CEN) on developmental timing and shoot and spike morphologies has been reported (Bi et al., 2019). No previous study has been conducted for the function of RADL TFs in wheat. In the current study, the expression of RADL TFs was different across the cultivars, and future study is necessary to reveal whether any genetic variation is present at the cultivar level of this gene family. Another top up-regulated abundantly expressed PLAC8 family protein can have conserved biochemical function due to its conserved core domain; however, specific functions of these family proteins are still unclear. In Arabidopsis, only PLAC8 domain-containing protein AtPCR and similar proteins in rice and other organisms (Song et al., 2011) are implicated in cadmium resistance. PLAC8-containing proteins can also control cell size and number (Frary et al., 2000) in plant. It is reported in yeast that cadmium tolerance can involve DNA repair (Di Vietro et al., 2014). In this study, as the PLAC8 family gene was up-regulated under N stress and high abundance was detected in a high NUE cultivar, it can be predicted that this gene may play an important role in N stress tolerance. Similarly, in the grain tissue of Mace, plasma membrane ATPase was identified as one of the top up-regulated DEGs (Supplementary Table 7), known to be induced in a condition that requires a greater transport activity and plays an important role in nutrient uptake (Janicka-Russak, 2011). Overexpression of plasma membrane ATPase is also associated with cadmium stress tolerance (Di Vietro et al., 2014). Another top up-regulated abundantly expressed DEG in Mace is a homolog of Arabidopsis (AT3G16150) K+-dependent L-asparaginase, which is associated with efficient metabolism of L-Asn under high metabolic demand of N (Bruneau et al., 2006). Its homolog in model legume *Lotus japonicus* has been reported to be involved in N remobilization and seed production (Credali et al., 2013). Alpha-galactosidase that highly up-regulated under N stress is a homolog of Arabidopsis AT5G08370, which plays an important role in leaf development by loosening and expanding cell wall (Chrost et al., 2007). It is also reported that alpha-galactosidase can contribute in completing energy-dependent senescence process and stress response in spite of severe decline in photosynthesis by maintaining the steady state of sugar supply through breakdown of wall polysaccharide (Pandey et al., 2017). To conclude, the above-mentioned genes, notably RADIALIS-like TFs, PLAC8 family proteins that are not characterized in wheat yet can be potential candidates to improve NUE and tolerance to N stress.

## Conclusion

Identification of DEGs across bread wheat genotypes with contrasting stress tolerance facilitates a better understanding of the genetic bases of N stress tolerance. Here, the RNA-seq analysis using second leaf and grain tissues of low and high N treated wheat plants demonstrated that gene transcripts involved in lipid biosynthesis, transmembrane transport, cell communication, and small molecule biosynthesis were abundantly expressed in low NUE cultivars under N stress. Higher expression of these genes will enable low-NUE genotypes to thrive under stress conditions. The abundance of N metabolism-related genes in low NUE cultivars also contributes to N stress adaptation. The DEGs among the three cultivars showed variation in the magnitude of change in the expression, which indicates varying degrees of tolerance to N stress. Genes that were differentially expressed between low and high N treatments can also be indirectly involved in N metabolism. The DEGs across genotypes provide an understanding of how differently wheat genotypes encounter the N stress and how they adapt. Common N responsive genes across cultivars indicated that these genes are involved in common pathways under N stress. Moreover, the top DEGs with high expression in a high NUE cultivar would be the potential candidates to be explored for improving wheat NUE.

## Data Availability Statement

The RNA-seq data has been deposited in the NCBI. Submission details: NCBI Sequence Read Archive (SRA) submission: SUB7918524.

## Author Contributions

NS carried out the entire experiment, analysis, and writing up. SI contributed to experimental management and writing. AJ contributed to experimental design and data interpretation. RY, MS, and JZ contributed in carrying out laboratory and glasshouse experiment. WM supervised the study and contributed to writing. All authors contributed to the article and approved the submitted version.

## Conflict of Interest

The authors declare that the research was conducted in the absence of any commercial or financial relationships that could be construed as a potential conflict of interest.

## Footnotes

1 https://www.bioworld.com/productinfo/3_43_288_690/126289/Hoagland-Medium-Nitrogen-Free.html

2 http://bioinformatics.sdstate.edu/go/

3 http://kobas.cbi.pku.edu.cn/

4 http://www.genome.jp/kegg/

5 https://string-db.org/

6 https://software.broadinstitute.org/morpheus/

## Supplementary Material

Supplementary Figure 1 KEGG enrichment pathways under N stress in three wheat cultivars Mace, Spitfire, and Volcani. **(A)** Mace leaf tissue; **(B)** Spitfire leaf tissue; **(C)** Volcani leaf tissue; **(D)** Mace grain tissue; **(E)** Spitfire grain tissue; **(F)** Volcani grain tissue.

Supplementary Table 1 Detailed of RNA-seq data and read mapping.

Supplementary Table 2 Summary of RNA-seq data and read mapping.

Supplementary Table 3 Common differentially expressed genes (DEGs) between leaf and grain tissue independent of developmental stage and genotype.

Supplementary Table 4 Consistently expressed DEGs between 0 and 10 DPA in second leaf tissue of of wheat cultivars Mace, Spitfire, and Volcani.

Supplementary Table 5 Top significantly up-regulated DEGs in the second leaf tissue of wheat cultivars Mace, Spitfire, and Volcani.

Supplementary Table 6 Top significantly down-regulated DEGs in the second leaf tissue of wheat cultivars Mace, Spitfire, and Volcani.

Supplementary Table 7 Top significantly up-regulated DEGs in the grain tissue of wheat cultivars Mace, Spitfire, and Volcani.

Supplementary Table 8 Top significantly down-regulated DEGs in the grain tissue of wheat cultivars Mace, Spitfire, and Volcani.

Supplementary Table 9 List of highly connected down-regulated DEGs in photosynthesis-antenna proteins.

Supplementary Table 10 List of highly connected down-regulated DEGs in photosynthesis.

Supplementary Table 11 List of highly connected down-regulated DEGs in steroid biosynthesis.
